# Co-pyrolysis of chicken manure with tree bark for reduced biochar toxicity and enhanced plant growth in *Arabidopsis thaliana*

**DOI:** 10.1038/s41598-024-62468-3

**Published:** 2024-06-17

**Authors:** A. Lataf, I. Pecqueur, M. Huybrechts, R. Carleer, F. Rineau, J. Yperman, A. Cuypers, D. Vandamme

**Affiliations:** 1 Analytical and Circular Chemistry, IMO, Centre for Environmental Sciences, Hasselt University, Agoralaan Building D, 3590 Diepenbeek, Belgium; 2 Environmental Biology, Centre for Environmental Sciences, Hasselt University, Agoralaan Building D, 3590 Diepenbeek, Belgium

**Keywords:** Co-pyrolysis, Chicken manure, Tree bark, Plant stress, Plant growth, *Arabidopsis thaliana*, Biochemistry, Plant sciences

## Abstract

Co-pyrolysis of chicken manure with tree bark was investigated to mitigate salinity and potentially toxic element (PTE) concentrations of chicken manure-derived biochar. The effect of tree bark addition (0, 25, 50, 75 and 100 wt%) on the biochar composition, surface functional groups, PTEs and polycyclic aromatic hydrocarbons (PAH) concentration in the biochar was evaluated. Biochar-induced toxicity was assessed using an in-house plant growth assay with *Arabidopsis thaliana*. This study shows that PTE concentrations can be controlled through co-pyrolysis. More than 50 wt% of tree bark must be added to chicken manure to reduce the concentrations below the European Biochar Certificate-AGRO (EBC-AGRO) threshold. However, the amount of PAH does not show a trend with tree bark addition. Furthermore, co-pyrolysis biochar promotes plant growth at different application concentrations, whereas pure application of 100 wt% tree bark or chicken manure biochar results in decreased growth compared to the reference. In addition, increased plant stress was observed for 100 wt% chicken manure biochar. These data indicate that co-pyrolysis of chicken manure and tree bark produces EBC-AGRO-compliant biochar with the potential to stimulate plant growth. Further studies need to assess the effect of these biochars in long-term growth experiments.

## Introduction

The world population is expected to grow to 10.4 billion in 2100^1^. This growing population leads to an increasing food demand while putting pressure on agricultural activities that face significant challenges due to climate change. Therefore, new cultivation materials are highly needed for sustainable and more efficient crop production. Current agricultural practices exert pressure on our environment by increasing soil degradation and decreasing soil health via tillage, pesticide and fertilisers use and mining activities^2,3^. Moreover, the production and use of nitrogen fertilisers contribute to greenhouse gas (GHG) emissions^4,5^. Biochar, a carbon-rich material produced through the thermal conversion of various biomass streams under anoxic conditions (pyrolysis), has the potential to help in alleviating these problems. At first, biochar’s persistent character can help reducing carbon dioxide emissions by long-term storage of carbon in the soil^6^. In addition, biochar can increase the plant nutrient availability, reduce nutrient leaching and increase microbial activity. As such, it increases plant growth and decrease the need for pesticide and fertiliser use^7^. Multiple studies already reported positive effects of biochar addition on plant growth^7–11^.

In regions with a high livestock density, like Flanders, pyrolysis can be an interesting waste management technique for livestock manure. Chicken manure is considered the largest nitrogenous livestock waste in this region, with approximately 600,000 tonnes of biomass generated annually^12^. Only a fraction of this manure (170 kg N/ha) can be applied as organic soil fertiliser because of the Flemish manure decree^13^. This results in a regional manure surplus and the need to process and export this product to other regions. Besides, manure can contain different pathogens and be a significant source of antibiotic residues^14,15^. Previous research has already indicated that manure-derived biochar is pathogen-free, and the associated thermal treatment causes the destruction of a wide range of pharmaceuticals^14,16^. Furthermore, Kimani et al. showed that poultry litter biochar increases rice yield and nitrogen use efficiency^17^. Also, positive effects of manure-derived biochar on the growth of lettuce, cherry tomato, maize, bean and *Jatropha curcas* were reported^10,18–20^. However, a potential drawback of biochar addition is the accumulation of toxic compounds like polycyclic aromatic hydrocarbons (PAHs) and potentially toxic elements (PTEs; Pb, Cd, Cu, Ni, Hg, Zn, Cr and As) in the soil^21,22^. The results of our previous study showed that pyrolysis of chicken manure results in biochars with a high concentration of Zn and Cu^23^. To mitigate the risk of biochar-induced toxicity, the European Biochar Certificate (EBC) proposed a maximal threshold of these elements (Cu: 100 mg/kg and Zn: 400 mg/kg)^24^. A possible way to reduce the amount of PTEs and PAHs is through co-pyrolysis with other feedstocks with less nutrient contents. As such, it was evidenced before that co-pyrolysis of PTE-rich sewage sludge with willow reduced the PTE concentration and the phytotoxicity of the resulting biochar^25^. In this, regard, tree bark is an interesting and regional wood by-product because of its biomass abundance and desirable characteristics^26^. This stream is characterised by its high carbon and lignin concentration and low PTE concentrations, as evidenced by our previous study. As such, this leads to carbon-rich biochars with low PTE amounts and is therefore an ideal biomass to mix with chicken manure in co-pyrolysis^23,27^.

To our knowledge, the effect of biomass ratios on the toxicity of the resulting biochar has not been investigated yet. Therefore, this study aims to investigate the effect of co-pyrolysis of different chicken manure-tree bark ratios on the biochars’ (phyto)toxicity as well as its growth promoting characteristics. The biochars were produced in a pilot-scale rotary kiln at 450 °C and characterised using elemental analysis, conductively coupled plasma-optical emission spectroscopy (ICP-OES) and Fourier-transformed- infrared (FT-IR) spectroscopy. The biochar toxicity in terms of PAHs and PTEs was evaluated, and the phytotoxicity and growth promotion were assessed using an in-house plant growth assay with *Arabidopsis thaliana* as a model plant.

## Materials and methods

### Biomass collection, pre-treatment and pyrolysis experiment

Chicken manure (CM) was collected from a local farm, and tree bark (TB) was supplied by Agaris (*Pinus maritima*)*.* The feedstocks were dried for 14 h at 105 °C, shredded below 2 mm, and blended in the desired mass ratios on a dry weight basis (TB-CM ratio: 100-0, 75-25, 50-50, 25-75, 0-100) before pelletisation. The specific mass ratios are described in Table 1 with the corresponding acronyms for the feedstock blends and resulting biochar. The pelletisation experiments were conducted in a mobile pelletiser (KL400) with a Ø 6 mm-pellet die. Milli-Q water was added to facilitate the throughput of pellets, and no other additives were used. After pelletisation, the pellets were dried at 105 °C for 14 h. Pyrolysis was carried out in a modified rotary kiln reactor at 450 °C as described in our previous publication^16^.

**Table 1:** The coding convention of the TB-CM feedstock blends and corresponding biochar used in this study.

TB-CM ratio	Feedstock blend	Biochar
100-0	BM-100-0	BC-100-0
75-25	BM-75-25	BC-75-25
50-50	BM-50-50	BC-50-50
25-75	BM-25-75	BC-25-75
0-100	BM-0-100	BC-0-100

### The analysis of the feedstock blends and the resulting biochars

The thermal behaviour of the feedstock blends (5–10 mg) was analysed by thermogravimetric analysis (TGA, Q500 TA instruments) according to the same procedure described in our previous publication^16^. In short, the sample was subjected to the following temperature program: (1) temperature ramp from 25 to 600 °C in N_2_ (20 °C/min), (2) gas flow was switched from N_2_ to O_2_, (3) isothermal step at 600 °C in O_2_ and (4) temperature ramp from 600 to 900 °C in O_2_.

The elemental analysis on feedstock blends and biochar (triplicate; 2–4 mg) (total carbon (TC), H, N, S) was carried out using the FlashEA 1112 series elemental analyser^16^. The sample’s O content was calculated by difference (O (wt%) = 100 − TC − H − S − N − ash). The ash content (triplicate; 0.5–1 g) was measured using a muffle furnace (B150, Nabertherm, Lilienthal, Germany) at 575 ± 25 °C for 3 h^23^.

FT-IR was carried out on all biomass blends and biochar samples using a Vertex 70 Spectrometer (DTGS detector) and attenuated total reflection (ATR) accessory (diamond crystal) (Bruker, Billerica, USA). Biochar absorbance was measured from 600 to 4000 cm^−1^, with a spectral resolution of 4 cm^−1^. The spectra were normalised (standard normal variate) and baseline corrected (baseline type: rubber) using the Quasar software version 1.2.0. (https://quasar.codes/)^28^.

The pH and electrical conductivity (EC) (mS/cm) were measured in triplicate after a 24-h extraction in Milli-Q water (solid:liquid ratio 1:10)^16^. The macronutrient (P, K, Mg and Ca) and PTE (Pb, Cd, Cu, Ni, Hg, Zn, Cr and As) concentrations of the feedstock blends and biochars were analysed through ICP-OES (Perkin Elmer Optima 8300) after a microwave-assisted two-step digestion (Milestone Ethos) with concentrated nitric acid (HNO_3,conc_; 69 wt% p.a.; Suprapur; Merck) and hydrogen peroxide (H_2_O_2_; 30 wt% p.a.; Supelco, Merck). In the first step, 7.0 mL HNO_3,conc_ and 3.0 mL H_2_O_2_ were added to the sample (100 mg) in Teflon vessels. Secondly, 3.0 mL HNO_3,conc_ and 2.0 mL H_2_O_2_ were added to the vessels. The microwave digestion was temperature controlled (220 °C, 20 °C/min with a holding time of 15 min). The digested samples were filtered and diluted with Milli-Q water to 50.00 mL. The PTE retention (%) was calculated using the same equation used in our previous study to assess secondary pollution^23^.

The total amount of 16 EPA PAHs (∑16 EPA PAHs) in biochar (1 g) was determined according to the International Biochar Initiative (IBI) method “Semi-volatile Organic Compounds by Gas Chromatography/Mass Spectrometry (GC–MS)” after Soxhlet extraction with 100% toluene (99.5%, AnalaR NORMAPUR®, VWR Chemicals) as extracting solvent (US EPA 8270e) (US EPA, 2018). A clear description of the complete method can be found in our previous publication^23^.

### Plant growth assay and biochar-induced stress assessment

*Arabidopsis thaliana Col-0* wild-type seeds (Nottingham Arabidopsis Stock Centre (NASC)) were surface sterilised with 70 vol% ethanol and rinsed several times using sterile dH_2_O before being stored for 2 nights at 4 °C to allow imbibition and synchronise germination. Subsequently, seeds were sown in 96-well plates (Greiner Bio-One) with ¼ Murashige and Skoog (MS) medium (referred to as reference growth medium) and exposed to the different biochar blends BC-100-0, BC-75-25, BC-50-50, BC-25-75 and BC-0-100 at the following biochar concentrations: 0.00 wt%, 0.05 wt%, 0.10 wt%, 0.25 wt% and 0.50 wt% (one 96-well plate per condition). Seedlings were grown with a photoperiod of 12 h, a day/night temperature of 22/18 °C and a relative humidity of 65%. Photosynthetic active radiation (PAR) was approximately 170 μmol m^2^ s^−1^ at the plant level and simulated using the GreenPower LED modules (Phillips) consisting of blue-, red- and far-red led modules. Four seedlings per sample and 8 biological replicates per condition were snap-frozen with liquid nitrogen seven days after sowing and stored at -80 °C for flow cytometry (FCM) analyses. Growth parameters like fresh weight and root length were determined of five seedlings per condition ten days after sowing.

An in-house screening platform (plant growth assay) based on FCM analysis with the CyStain PI Absolute kit (Sysmex-Partec) was used to evaluate biochar-induced stress or growth promotion. Seedlings were chopped in 250 µL of nuclei extraction buffer and incubated for 1 min. Afterwards, the extract was filtered with a 50 µm CellTrics®. Subsequently staining solution consisting of 1 mL staining buffer, 6 µL PI and 3 µL RNAse A per sample was added to stain the DNA. After at least 1 h of incubation at 4 °C, samples were measured using a CyFlow Cube 8 Flow Cytometer (Sysmex-Partec). A 488 nm laser was used to excite the nuclei, and the forward scatter and PI fluorescence intensity (FL-2 channel, 580/30 nm) were measured. Data analysis was performed with the FCS Express 4 software (De Novo Software; https://denovosoftware.com/full-access/download-landing/), and endoreplication indices for plant growth (EI_growth_) and phytotoxicity (EI_defence_) were calculated according to Cuypers et al.^29^.

To determine the leaching and adsorption of elements by biochar, biochars (BC-100-0, BC-75-25, BC-50-50, BC-25-75 and BC-0-100) were added to the medium at the same concentration levels as used for the plant growth experiments (0.00 wt%, 0.05 wt%, 0.10 wt%, 0.25 wt% and 0.50 wt%). Afterwards, the medium was stored under similar conditions as the plant growth experiments for ten days. Subsequently, the medium was filtered to remove the biochar, acidified with HNO_3,conc_ and ICP-OES was used to determine the dissolved K^+^, Na^+^, Ca^2+^, Mg^2+^, Zn^2+^, Cu^2+^ and total P.

### Statistics

Statistical analyses were executed with R studio version 4.2.2 (https://posit.co/download/rstudio-desktop/)^30^. Outliers were identified with the Grubbs test and deleted before further analysis. Normality and homoscedasticity of the data were checked with the Shapiro–Wilk test and the Barlett test, respectively. If normality was not fulfilled, the data's square root, inverse, exponential and logarithmic transformations were tested. If the data (or transformed data) met the assumptions, one-way ANOVA was used to identify significant differences between the different concentrations used within the same biochar type. When the data did not meet the assumptions, a nonparametric Kruskal–Wallis with post hoc Wilcoxon rank-sum exact test was executed. Furthermore, Pearson correlation coefficients were calculated after a visual inspection of normality with a qq-plot.

### Ethics approval

The plant experiments were in accordance with relevant institutional, national, and international guidelines and legislations. Additionally, experiments do not violate the IUCN policy statement on Research Involving Species at Risk of Extinction and Convention on the Trade in Endangered Species of Wild Fauna and Flora.

## Results and discussion

### Biochar yield and characteristics

**Figure 1:**
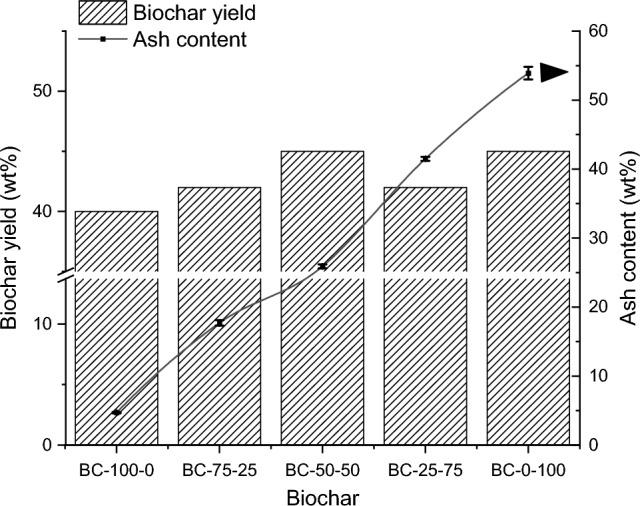
The biochar yield (left Y-axis) and ash content (right Y-axis) of the different produced *co*-pyrolysis biochars (TB-CM).

Figure 1 shows the biochar yield and ash content obtained after the pyrolysis experiment. The biochar yield varied between 40 (BC-100-0) and 45 wt% (BC-0-100), and the ash content varied between 4.7 (BC-100-0) and 53.9 wt% (BC-0-100). The relatively high biochar yield of BC-100-0 compared to woody and agricultural biochars in general (20-30 wt% biochar yield at a pyrolysis temperature of 450 °C) resulted from the high fixed carbon content of BM-100-0 (40 wt% on a dry and ash-free basis) (Table S1). In contrast, the biochar yield of BC-0-100 was more related to its high ash content (Fig. 1)^31^. The biochar yield of BC-75-25, BC-50-50 and BC-25-75 fluctuated between 42 and 45 wt% but within the pyrolysis experimental error (as a standard deviation: 4 wt%)^23^. When looking more closely at the volatile matter (57–62 wt% dm) (Table S1) and the thermogram (Fig. S1), there seems to be no large difference between the different feedstock blends around the pyrolysis temperature used (450 °C). Therefore, a similar biochar yield can be expected for the corresponding biochar. Although the biochar yield of the co-pyrolysis biochars was in a similar range, a difference in composition and biochar characteristics can be expected because of the increasing mineral fraction with CM addition.

Table 2 shows the co-pyrolysis biochars' elemental composition (TC-HNO), TC/N ratio, ash content, macronutrients (P, K, Ca and Mg), pH and EC. Total carbon (TC) content ranged from 37 wt% (BC-0-100) to 78 wt% (BC-100-0) and was negatively related to CM addition. This was substantiated by the increasing ash content with CM addition (Fig. 1) and also visible in the macronutrient concentration; CM addition caused significantly more P, K, Ca and Mg in the biochars, which increases their fertilisation potential. Similar trends were already observed for biochar produced through co-pyrolysis of pig manure and lignin^32^. The biochars’ P, K, Ca and Mg concentrations varied respectively between 0.4–28.3 mg/g, 2.3–34.8 mg/g, 6–182 mg/g and 1.67–14 mg/g. The Ca concentration in the produced biochars reached a higher concentration than the other macronutrients. This suggests that the biochars can potentially be used as liming agents^33^. Biochar samples BC-100-0 and BC-75-25 demonstrated a high C/N ratio (> 35), indicating the potential to stimulate N mineralisation through microbial activity. In contrast, BC-25-75 and BC-0-100 had a low C/N ratio (< 20), favouring their use as a fertiliser. BC-50-50 showed an intermediate C/N ratio (28.2) which is believed to cause an optimum equilibrium state between N mineralisation and immobilisation^34^. The biochars showed an alkaline nature with a pH varying between 7.96 and 10.2. There seemed to be a stabilising effect around a pH of 10 for BC-25-75 and BC-0-100. This could be due to the acid-buffering effect of biochar induced by inorganic carbon (carbonate). Table S1 indeed shows that BM-25-75 and BM-0-100 have an increased carbonate concentration (4 wt% dm) compared to the other feedstock blends (< 1 wt% dm) based on the TGA thermogram. Our previous study highlighted the importance of the Ca and carbonate concentrations for the pH and acid-buffering capacity, respectively^23^. The addition of CM also caused an increase in the EC between 0.3 (BC-100-0) and 8.57 mS/cm (BC-0-100), which means the biochar has the potential to release more macronutrients into its environment. This was evaluated by incubating the biochars for 10 days in the reference growth medium at different concentrations (0.05, 010, 0.25 and 0.50 wt%). The concentration of the different leached nutrients (Na^+^, K^+^, Mg^2+^, Ca^2+^ and total P) in the supernatant was analysed (Figs. S3–S5). The results indicated a high leaching of Na^+^ and K^+^ into the reference growth medium, especially for BC-25-75 and BC-0-100 (Fig. S3). For Na^+^, the concentration even increased by approximately one order of magnitude at a biochar concentration of 0.50 wt%. The Mg^2+^ concentration in the supernatant increased with an increasing biochar concentration. The Mg^2+^ concentration in the growth medium was also controlled by the biochar’s total Mg content, which increased with the increasing CM addition in the biochar’s feedstock blend. In contrast, the leaching of Ca^2+^ showed a different trend; the biochar with the highest Ca content resulted in a low concentration in the growth medium and was generally higher for the biochars with the lowest Ca content. Furthermore, a decreasing and complete depletion of P in the growth medium was found with an increased biochar concentration of BC-100-0. This depletion of P in soils was also found in previous research^35^. This shows that the leaching of elements from biochars depends on different factors and is very complex.

**Table 2:** The co-pyrolysis biochars’ elemental composition (TC-HNO), the TC/N, O/C and H/C ratios, ash content, macronutrients (P, K, Ca, Mg), pH and electrical conductivity.

Biochar	Elemental composition				TC/N	Molar O/C	Molar H/C	Macronutrients				pH	EC
	TC	H	N	O by diff				P	K	Ca	Mg		
	wt%	wt%	wt%	wt%	–	–	–	mg/g	mg/g	mg/g	mg/g	–	mS/cm
BC-100-0	74.4 (0.6)	2.76 (0.04)	0.62 (0.05)	17.5 (0.8)	120 (9)	0.176 (0.008)	0.446 (0.007)	0.400 (0.005)	2.30 (0.01)	6 (0.2)	1.67 (0.04)	7.96 (0.06)	0.3 (0.1)
BC-75-25	61.5 (0.7)	2.5 (0.04)	1.51 (0.07)	17 (1)	41 (2)	0.20 (0.02)	0.488 (0.009)	7.2 (0.1)	10.0 (0.3)	55 (3)	4.88 (0.04)	8.8 (0.03)	1.139 (0.003)
BC-50-50	55.6 (0.9)	2.26 (0.06)	1.97 (0.05)	14 (1)	28.2 (0.9)	0.19 (0.02)	0.49 (0.02)	12.1 (0.2)	15.5 (0.3)	82.0 (0.5)	7.2 (0.1)	8.98 (0.01)	3.005 (0.007)
BC-25-75	44.5 (0.6)	1.94 (0.05)	2.56 (0.08)	9 (1)	17.4 (0.6)	0.16 (0.02)	0.52 (0.02)	21.3 (0.9)	26 (0.9)	149 (2)	11.5 (0.1)	10.11 (0.01)	6.27 (0.04)
BC-0-100	36.6 (0.7)	1.67 (0.02)	2.94 (0.08)	5 (2)	12.4 (0.4)	0.10 (0.03)	0.55 (0.01)	28.3 (0.7)	34.8 (0.7)	182 (2)	14.0 (0.3)	10.2 (0.1)	8.57 (0.04)

The values represent the average (standard deviation).

### The co-pyrolysis biochar surface functionalities

**Figure 2:**
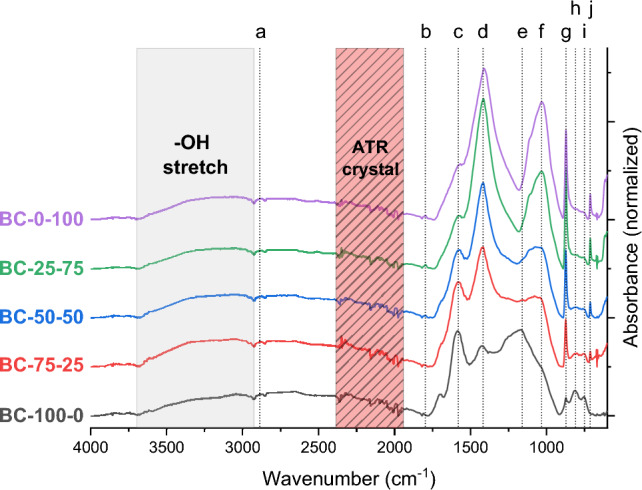
The normalised and baseline corrected ATR-FTIR spectra (absorbance mode) of TB-CM-based co-pyrolysis biochars.

Figure 2 shows the FT-IR spectra of the co-pyrolysis biochars and their corresponding starting materials between 600 and 4000 cm^−1^. The FT-IR spectrogram of the corresponding feedstock blends can be found in Figure S2. The first broad peak in the grey region (approx. 3000–3600 cm^−1^) corresponds to –OH stretching from different O-rich functional groups (–COOH, –OH, …). This peak was more present for the feedstock blends than the biochars because of the partial thermal degradation of O-rich functionalities. The two peaks at 2924 and 2851 are respectively associated with the asymmetric and symmetric stretching of alkyl (–CH_x_) groups and are also found in all feedstock blends (Fig. S2). After pyrolysis, these peaks were inexistent and were substituted by a broader peak around 2887 cm^−1^ (a), representing alkylated surface groups. The peaks between 1700 and 1800 cm^−1^ (b) were small or existed as a shoulder peak in the spectra and can be assigned to the vibration of C=O^36^. The peak in the 1600 cm^−1^ region (c) is associated with C=C structures but can also correspond to the N–H stretching of amide groups^37,38^. This might be a plausible explanation as the biochars showed an increasing N content with CM addition^37,38^. However, this peak became a shoulder peak with CM addition, which contradicts that the peak originated from N species. Peaks around this region can also be attributed to -OH in-plane bending, C=O, and other oxygenated functionalities^39^. This could be substantiated because of the decreasing O content in the biochar with the increasing CM addition (Table 2). Interestingly the peaks around 1428 (d) and 872 (g) cm^−1^, associated with carbonate (CO_3_^2-^), existed in the feedstock blends BM-75-25, BM-50-50, BM-25-75 and BM-0-100 and their corresponding biochars. The relative intensity of the peak at 872 cm^−1^ became more intense with the addition of CM, which further confirms that this peak can be attributed to CO_3_^2−^ (Table S1)^40^. Besides CO_3_^2−^, the peaks 1428 (d) and 1034 (f) cm^−1^ can also be attributed to, respectively, C=C stretching and C–O–C asymmetric stretch^31^. For BC-100-0, these peaks did not exist, and only a broad band at 1165 (e) cm^-1^ was found that can be attributed to C–O stretching, as was also observed for tree bark-based biochar^41^.

### Biochar toxicity assessment

#### Implications of PTEs during and after co-pyrolysis

As was stated by earlier studies, a major drawback of manure and sludge pyrolysis is the accumulation of PTEs in the biochar^22,25,42^. Some of these elements (Zn and Cu) are essential micronutrients for plants but become highly toxic at elevated concentrations. Other PTEs, such as Cr, are not beneficial for plants and induce toxicity. Table 3 shows the different biochars' Zn, Cu and Cr concentrations and their retention from feedstock after pyrolysis into biochars. The Zn, Cu and Cr concentrations varied respectively between 44.7–635 mg/kg, 66–184 mg/kg and 17.4–70 mg/kg. While the Zn retention in all biochars after pyrolysis was nearing 100 wt% (94 ± 6%), its Cu retention was lower (70 ± 8 wt%), which suggests that part of the Cu is transferred to the gas phase during pyrolysis. In contrast, the Cr retention reached up to 273 wt% (BC-100-0), which can be explained by Cr contamination from the stainless steel (type 316) reactor. The biochars BC-75-25, BC-50–50 and BC-25–75 showed a Cr retention between 102 and 129 wt% (113 ± 22 wt%), not significantly different from 100 wt% (*p* = 0.11). On the other hand, BC-0-100 showed a partial transfer of Cr to the gas phase through its low retention in the biochar (43 wt%). Only the biochars BC-25-75 and BC-0-100 showed a Zn and Cu concentration above the EBC-AGRO threshold (400 (Zn) and 100 (Cu) mg/kg). This means that 50 wt% of TB must be added to produce biochar that complies with this threshold. However, these PTEs need to be bioavailable to induce toxic effects in plants. In the case of the biochars used in this study, there was no additional leaching of Zn^2+^ into the reference growth medium after 10 days of biochar incubation, regardless of the biochar concentration and biochar type (Fig. S6). There was even a decreasing trend with increasing biochar concentrations, evidencing some uptake of this element by the biochar. The reduction in the bioavailability of Zn by biochar was already evidenced^43^. The Cu and Cr concentrations in all (biochar-amended) growth media was not reported because both elements were not detected (Cu < 0.25 mg/L; Cr < 0.05 mg/L). This means that although the Zn^2+^ and Cu^2+^ concentrations in the biochars BC-25-75 and BC-0-100 exceeded the EBC-AGRO threshold, these biochars are not a source of PTEs in the current experiment. However, the long-term leaching effects of PTEs from biochar should be further assessed under various weathering conditions.

**Table 3:** The Zn, Cu and Cr concentrations and retention of the different biochars.

Biochar	Zn	Zn retention	Cu	Cu retention	Cr	Cr retention
	mg/kg	wt%	mg/kg	wt%	mg/kg	wt%
BC-100–0	44.7 (0.4)	–	n.d	–	70 (10)	273 (87)
BC-75–25	195 (5)	94 (3)	66 (2)	63 (2)	27.3 (0.8)	102 (16)
BC-50–50	294 (5)	92 (5)	94 (1)	68 (5)	28 (3)	102 (27)
BC-25–75	**490 (17)**	90 (3)	**146 (6)**	71 (3)	40 (10)	129 (54)
BC-0–100	**635 (17)**	101 (6)	**184 (4)**	81 (4)	17.4 (0.7)	43 (18)

The standard deviation of the Zn and Cu retention is calculated based on the error propagation theorem. The bold numbers exceed the EBC-agro limits. Other potentially toxic elements (Ni: < 10 mg/kg dm; Pb: < 10 mg/kg dm. Cd: < 1 mg/kg dm, Hg: < 1 mg/kg dm and As: < 10 mg/kg dm) were below the detection limit. n.d.: not detected. The values represent the average (standard deviation).

#### The co-pyrolysis biochars’ polycyclic aromatic hydrocarbon (PAH) concentration

**Figure 3:**
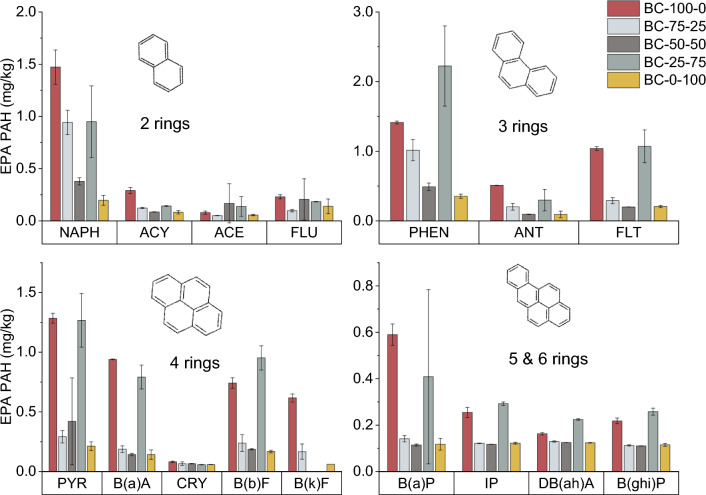
The individual PAHs of the ∑16 EPA PAHs for the co-pyrolysis biochars. NAPH: naphthalene, ACY: acenaphthylene, ACE: acenaphthene, FLU: fluorene, PHEN: phenanthrene, ANT: anthracene, FLT: fluoranthene, PYR: pyrene, B(a)A: benzo(a)anthracene, CRY: chrysene, B(b)F: benzo(b)fluoranthene, B(a)P: benzo(a)pyrene, IP: indeno(1,2,3-cd)pyrene, DB(ah)A: dibenzo(a,h)anthracene, B(ghi)P: benzo(ghi)perylene. The bars represent the average, and the error bars represent the standard deviation.

In addition to PTEs, also PAHs could form a threat to crop growth^44^. Table 4 shows the ∑16 EPA PAHs and ∑16 BAP-equivalent concentration. The Σ16 EPA PAHs concentration varied between 2.17 (BC-0-100) and 8.9 (BC-100-0) mg/kg, and the ∑16 BAP-equivalent concentration varied between 0.288 (BC-50-50) and 1.02 (BC-100-0) mg/kg. BC-100-0 and BC-25-75 showed the highest ∑16 EPA PAHs and ∑16 BAP-equivalent concentrations, and it seems that the addition of 25 and 50 wt% CM to the TB caused a decrease in ∑16 EPA PAHs and ∑16 BAP-equivalent concentrations. An increase in the Σ16 EPA PAHs and ∑16 BAP-equivalent concentrations was observed for BC-25-75. This suggests that co-pyrolysis affects the Σ16 EPA PAHs concentration positively and negatively. Figure 3 shows the different ∑16 EPA PAHs constituents in the biochar. When having a closer look at the different ∑16 EPA PAHs constituents in BC-100-0 and BC-25-75, phenanthrene (PHEN), fluoranthene (FLT), pyrene (PYR), benzo(b)fluorene (B(b)F), benzo(a)anthracene (B(a)A) and benzo(a)pyrene (B(a)P) showed an increased concentration compared to the other biochars. The high B(a)P concentration in these biochars also contributed 48 – 58 wt% to the increased ∑16 BAP-equivalent concentration. The naphthalene (NAPH) concentration in the biochars initially decreased with increasing CM addition from 1.5 (BC-100–0) to 0.38 mg/kg (BC-50-50). For BC-25-75, the concentration increased to 0.9 mg/kg. Only a limited quantity (< 0.4 mg/kg) of each of the following PAHs was detected in all the biochars: acenaphthylene (ACY), acenaphthene (ACE), fluorene (FLU), anthracene (ANT), chrysene (CRY), indeno(1,2,3-cd)pyrene (IP), dibenzo(ah)anthracene (DB(ah)A) and benzo(ghi)perylene (B(ghi)P). However, DB(ah)A significantly contributed (16–44%) to the ∑16 BAP-equivalent concentration.

**Table 4:** The ∑16 EPA PAHs and ∑16 BAP-equivalent concentrations of the co-pyrolysis biochars are represented as average (standard deviation).

Biochar	∑16 EPA PAHs (mg/kg)	∑16 BAP-equivalent (mg/kg)
BC-100-0	**8.9 (0.1)**	1.02 (0.06)
BC-75-25	3.8 (0.3)	0.35 (0.03)
BC-50-50	2.7 (0.4)	0.288 (0.004)
BC-25-75	**8.4 (0.7)**	0.9 (0.4)
BC-0-100	2.17 (0.07)	0.29 (0.03)

The bold numbers exceed the EBC-agro limits. The values represent the average (standard deviation).

The formation and occurrence of PAHs during biomass pyrolysis still need to be fully understood. However, a previous study evidenced that the formation of some PAH constituents is affected by the interaction between cellulose, hemicellulose and lignin^45^. As the ratio of these compounds changed for the different feedstock blends, another interaction can be expected, potentially leading to an increased ∑16 EPA PAHs concentration. However, in the current pilot-scale set-up, the ∑16 EPA PAHs concentration in the biochar is also partly dependent on their condensation during biochar collection, as was proposed in a previous study^23^.

When combining the biochar toxicity data (PTEs, ∑16 EPA PAHs and ∑16 BAP-equivalent concentrations), up to 50 wt% CM can be added to the TB before pyrolysis to produce EBC-compliant biochar. In the case of BC-100-0, the high ∑16 EPA PAHs concentration resulted in non-compliance. Because of the complexity of PAH formation, this toxicity factor should continuously be assessed for different feedstock blends and feedstock ratios.

### Plant growth and toxicity assessment

**Figure 4:**
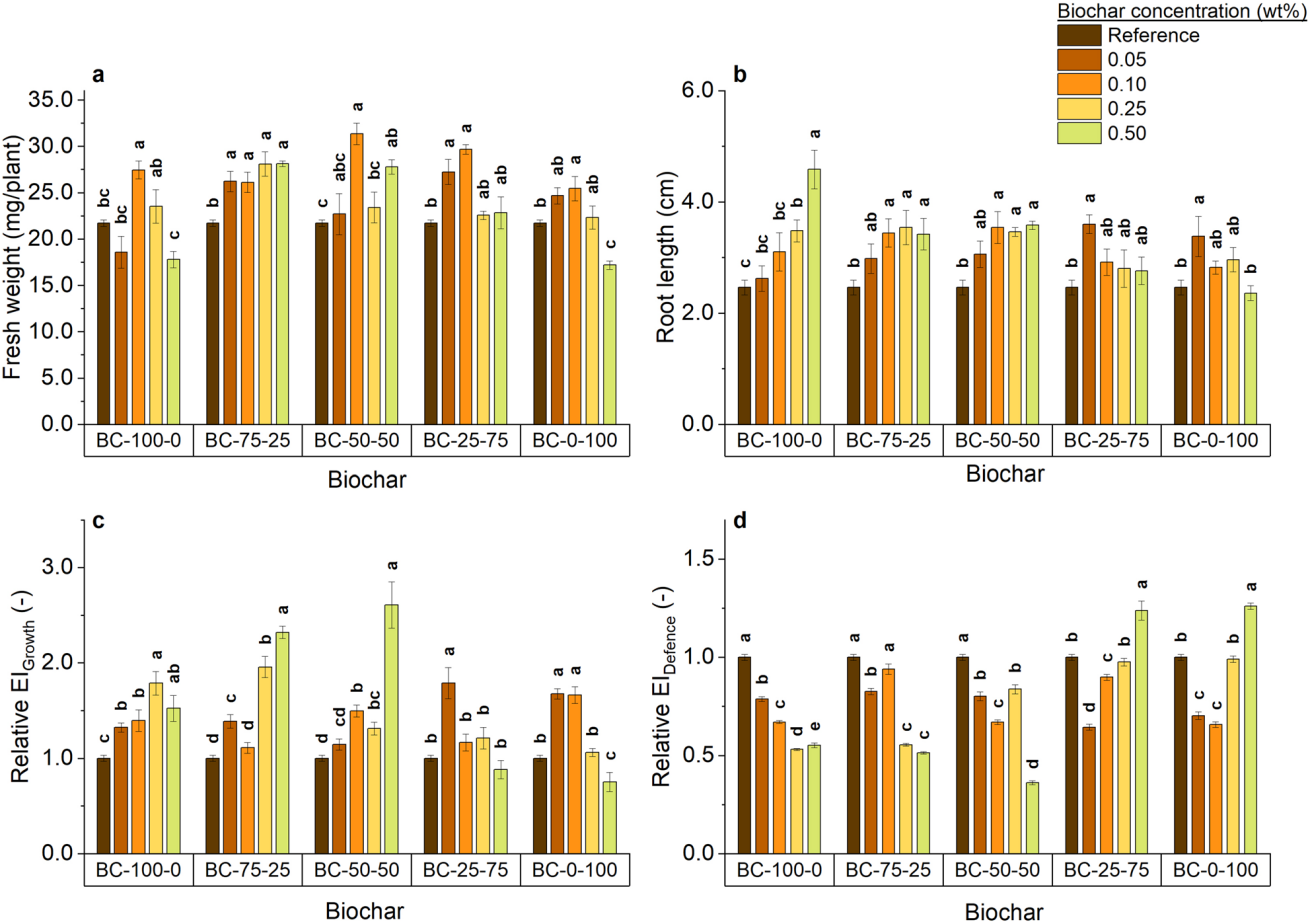
Biometric parameters and endoreplication indices (EI) of *A. thaliana* seedlings grown for 7 (EI determination) or 10 (biometric analyses) days in a 96-well plate with reference growth medium and treated with different biochars (BC-100-0, BC-75-25, BC-50-50, BC-25-75, BC-0-100) at different concentrations (0, 0.05, 0.10, 0.25, 0.50 wt%): (**a**) fresh weight, (**b**) root length, (**c**) relative EI_growth_, (**d**) relative EI_defence_. The bars represent the average of minimal five biological replicates, and the error bars represent the standard error. Significant differences within the same biochar are indicated with different letters (one-way ANOVA *p* < 0.05).

An in-house plant growth assay was used with *A. thaliana* as a model plant to assess biochar-induced phytotoxicity or its growth-promoting effects. Figure 4 shows the biometric parameters, i.e. plant fresh weight (Fig. 4a) and root length (Fig. 4b), as well as the relative EI_growth_ (Fig. 4c) as an indicator for plant growth promotion and the relative EI_defence_ (Fig. 4d) as an indicator for plant stress^29^. All biochars significantly affected the fresh weight (Fig. 4a) and root length (Fig. 4b) of *A. thaliana* seedlings compared to seedlings grown in the reference growth medium. However, this depends on the biochar blend used and the concentration applied. For each biochar, there is an optimal biochar concentration. In general, pure biochars, i.e. BC-100-0 and BC-0-100, had no positive effect on the fresh weight (except at 0.10 wt% application) and even significantly decreased plant fresh weight when 0.50 wt% BC-0-100 was added as compared to seedlings grown in the reference growth medium. A similar observation was seen for the biochar combinations with the higher CM addition (BC-50-50 and BC-25-75). On the other hand, the fresh weight of seedlings exposed to BC-75-25 increased compared to the seedlings grown in the reference growth medium, independent of the biochar concentration applied. Furthermore, the root length (Fig. 4b) of seedlings treated with BC-75-25 and BC-50-50 significantly increased for all biochar concentrations, except when 0.05 wt% was added. The 0.05 wt% concentration of BC-25-75 and BC-0-100 did increase the root length significantly. However, a decreasing trend in root length, down to the root length of the seedlings grown in the reference growth medium, was visible when the biochar concentrations increased. On the other hand, an increasing trend in root length was visible with rising biochar concentrations when seedlings were treated with BC-100-0, with a significantly longer root when 0.25 or 0.50 wt% was added to the reference growth medium. The latter could be explained by the P-deficient environment created by the amendment of BC-100-0 (Fig. S5). Phosphorus is a known macronutrient that plays an important role in plant growth and development and is involved in multiple cellular processes like photosynthesis and energy production^46^. Moreover, a previous study indicated that *A. thaliana* and other plants change their root architecture depending on the P concentration as part of their P acquisition strategy^47^. Furthermore, the importance of the P concentration for plant growth in our experiment is indicated by a trend towards significance for the correlation between the P concentration in the medium and the resulting fresh weight (r = 0.43; p = 0.068). In addition, biochar treatment with BC-100-0, BC-75-25 and BC-50-50 significantly increased EI_growth_ (Fig. 4c) and decreased EI_defence_ (Fig. 4d) levels in plants compared to the seedlings grown in the reference growth medium. The decrease in EI_defence_, an indicator of plant stress, in seedlings treated with BC-100-0 indicates that although the biochar contained a Σ16 EPA PAHs concentration that exceeded the EBC-AGRO threshold (Table 4), these PAHs do not cause PAHs-induced stress in *A. thaliana* seedlings in this set-up and further confirms that P deficiency in the medium with BC-100-0 is the main reason for the decreased growth. Based on the Σ16 EPA PAHs concentration of current biochars (Table 4), the maximal concentration of Σ16 EPA PAHs in the medium would be only 0.04 mg Σ16 EPA PAHs/L at the highest biochar concentration, in case all the PAHs leach out to the reference growth medium. This concentration is much lower than concentrations used to study PAH toxicity in *A. thaliana.* For example, multiple studies used phenanthrene to study the effect of PAHs on *A. thaliana* growth with concentrations ranging between of 17–222 mg/L^48–51^. Moreover, earlier research already evidenced that only a fraction of the Σ16 EPA PAHs (max. 16%) is released from (sewage sludge) biochar (Σ16 EPA PAHs: 1–14 mg/kg), with almost no release of 3 + rings PAHs^52^. Thus, much lower Σ16 EPA PAHs are expected compared to the maximal theoretical concentration (0.04 mg Σ16 EPA PAHs/L). A previous study evidenced acute PHEN-induced stress in *A. thaliana* at much higher concentrations (approx. 17 mg PHEN/L)^48^. This confirms that in the current set-up no PAHs-induced stress in *A. thaliana* is expected. However, in line with PTEs released from biochar, the persistence and toxicity of PAHs released from biochar should be further assessed under different weathering conditions and at different trophic levels.

Furthermore, for the biochars with more CM contribution, i.e. BC-25-75 and BC-0-100, a significant increase of the EI_defence_ concomitantly with no effect or a decrease in EI_growth_ were found in seedlings when treated with 0.50 wt%. This increased stress could be caused by the higher concentrations of K^+^ and Na^+^ found in the medium after biochar BC-25-75 or BC-0-100 incubation (Figure S3). This theory is confirmed by the significant correlations between K^+^ (r = 0.50)/ Na^+^ (r = 0.48) concentration in the medium and the relative seedling’s EI_defence_ values. Therefore, the positive effects of treatment with BC-75-25 and BC-50-50 observed regarding fresh weight, reduced plants stress (EI_defence_) and increased plant growth (EI_growth_) are believed to come from the right balance between the P and Na^+^/K^+^ concentration, as these correlate to the fresh weight and EI_defence_, respectively. The treatment with biochars BC-50-50 and BC-75-25 resulted in the highest P concentrations in the medium, which could also explain the increased plant growth. This indicates that co-pyrolysis can be a technique to promote plant growth through biochar engineering.

## Conclusions

In this study, the benefits of co-pyrolysis of CM and TB on the plant growth of *A. thaliana* were investigated*.* Our results showed that the biochar composition and toxicity could be controlled through co-pyrolysis. In case of biochar toxicity, up to 50 wt% of CM can be added to the TB without exceeding the EBC-AGRO PTE threshold in the resulting biochar. In the case of the PAHs concentration, no trend was observed, but no biochar, except BC-100-0 and BC-25-75, exceeded the EBC-AGRO threshold. All co-pyrolysis biochars increased the fresh weight and decreased plant stress independent of the used concentrations, except biochars originating from feedstock blends with a high concentration of CM. In contrast, biochars from the original feedstocks showed an increased stress response and/or a reduced plant fresh weight. This could be linked to the leached total P and Na^+^/K^+^ concentrations as these are correlated to growth and stress parameters. Therefore, the biological outcome can be strongly linked to the biochars’ composition. These results suggest that co-pyrolysis can be used to promote plant growth through biochar engineering by modifying the physicochemical properties that decrease the biochar toxicity, while maintaining its positive characteristics, hence improving plant growth. To confirm these results, further research should be carried out to investigate the long-term effects of co-pyrolysis biochar on plant growth and stress response.

### Supplementary Information

Supplementary Information.

## Data Availability

The datasets used or analysed during the current study are available from the corresponding author upon reasonable request.
