# Supplementary material for: Co-pyrolysis of chicken manure with tree bark for reduced biochar toxicity and enhanced plant growth in Arabidopsis thaliana
Source: Sci Rep. 2024 Jun 17;14:13956. doi: 10.1038/s41598-024-62468-3 (PMC11183055; doi:10.1038/s41598-024-62468-3)
Supplement: Supplementary file 1 — Supplementary Information. [file 41598_2024_62468_MOESM1_ESM.docx]

**Co-pyrolysis of chicken manure with tree bark for reduced biochar toxicity, enhanced plant growth and reduced stress response in *Arabidopsis thaliana*: Supplementary Materials**

A. Lataf^a^, I. Pecqueur^b^, M. Huybrechts^b^, R. Carleer^a^, F. Rineau^b^, J. Yperman^a^, A. Cuypers^b^, D. Vandamme^a*^

^a^ Hasselt University, Analytical and Circular Chemistry, IMO, Centre for Environmental Sciences, Agoralaan Building D, 3590 Diepenbeek, Belgium

^b^ Hasselt University, Environmental Biology, Centre for Environmental Sciences, Agoralaan Building D, 3590 Diepenbeek, Belgium

*Corresponding author, E-mail: dries.vandamme@uhasselt.be

Figure S1 - The TGA curves (20 °C/min) of the different TB-CM feedstock blends (20-900 °C)

Table S1– The TGA characteristics (moisture, volatile matter, fixed carbon, ash (600°C) and carbonate content) of the different TB-CM feedstock blends

| **Feedstock blend** | **Moisture**  **(wt%)** | **volatile matter (wt% dm)** | **Fixed carbon**  **(wt% daf)** | **Ash**  **(wt% dm)** | **Carbonate**  **(wt% dm)** |
| --- | --- | --- | --- | --- | --- |
| BM-100-0 | 6 | 58 | 40 | 3 | 0 |
| BM-75-25 | 7 | 60 | 34 | 9 | 1 |
| BM-50-50 | 5 | 62 | 29 | 13 | 1 |
| BM-25-75 | 6 | 58 | 28 | 19 | 4 |
| BM-0-100 | 7 | 57 | 24 | 26 | 4 |

Figure S2 - The normalised and baseline-corrected ATR-FTIR spectra (absorbance mode) of the different TB-CM feedstock blends

Table S2 – The calculated P, K, Mg and Ca retention in the co-pyrolysis biochars

| **Biochar** | **P retention** | **K retention** | **Ca retention** | **Mg retention** |
| --- | --- | --- | --- | --- |
|  | **%** | **%** | **%** | **%** |
| BC-100-0 | 108 (5) | 110 (3) | 83 (14) | 101 (3) |
| BC-75-25 | 95 (4) | 97 (3) | 101 (7) | 96.4 (0.9) |
| BC-50-50 | 96 (5) | 98 (5) | 96 (2) | 96 (4) |
| BC-25-75 | 99 (2) | 98 (3) | 101 (6) | 101 (1) |
| BC-0-100 | 94 (9) | 102 (3) | 94 (6) | 95 (7) |
| Average | 98 (26) | 101 (18) | 95 (35) | 98 (17) |





Figure S3 – The Na^+^ and K^+^ concentration in growth medium at different biochar amendments of the co-pyrolysis biochars





Figure S4 – The Mg^2+^ and Ca^2+^ concentration in growth medium at different biochar amendments of the co-pyrolysis biochars





Figure S5 – The total P concentration in growth medium at different biochar amendments of the co-pyrolysis biochars





Figure S6 – The Zn^2+^ concentration in growth medium at different biochar amendments of the co-pyrolysis biochars

Table S3 – The Pearson correlation coefficients between the biological parameters (plant fresh weight, root length, relative EI_defence_ and relative EI_growth_) and macro-and micronutrient (P, K, Ca, Mg, S, Na, Zn, Fe and Mn) concentrations in the growth medium. An asterisk indicates a trend toward significance (p < 0.1), and two asterisks indicate a significant difference (p < 0.05)

| **Concentration in the growth medium (mg/L)** | **Plant fresh weight (mg/plant)** | **Root length (cm)** | **Relative EI_defence_ (-)** | **Relative EI_growth_ (-)** |
| --- | --- | --- | --- | --- |
|  |  |  |  |  |
| P | 0.43* | -0.18 | -0.04 | 0.21 |
| K | -0.21 | -0.4* | 0.50** | -0.23 |
| Ca | 0.27 | 0.61** | -0.36 | 0.18 |
| Mg | 0.00 | -0.28 | 0.35 | -0.07 |
| S | -0.19 | -0.47** | 0.53** | -0.27 |
| Na | -0.21 | -0.38 | 0.48** | -0.2 |
| Zn | 0.26 | -0.02 | -0.19 | 0.07 |
| Fe | -0.28 | 0.37 | -0.26 | -0.04 |
| Mn | -0.27 | 0.16 | -0.13 | -0.15 |
